# Efficient Offline Compensation of In-Air Magnetic Flux for Accurate Characterization of Soft Magnetic Materials

**DOI:** 10.3390/s26154801

**Published:** 2026-07-28

**Authors:** Vittorio Bertolini, Marco Stella, Antonio Faba, Ermanno Cardelli

**Affiliations:** Engineering Department, University of Perugia, 06125 Perugia, Italy; marco.stella@unipg.it (M.S.); antonio.faba@unipg.it (A.F.); ermanno.cardelli@unipg.it (E.C.)

**Keywords:** soft magnetic materials, Epstein characterization, air magnetic flux compensation

## Abstract

**Highlights:**

**What is the main finding?**

- A simple technique for in-air magnetic flux compensation in Epstein Frame characterization of soft magnetic materials.

**What is the implications of the main finding?**

- Characterize soft magnetic materials with the Epstein Frame, avoiding errors due to the presence of in-air magnetic flux occurring at high magnetic flux density values without the necessity of additional components in the experimental setup or complex methods for the evaluation of the surface of the Epstein coil.

**Abstract:**

This paper proposes an efficient and easily applicable offline method to compensate for the air magnetic flux in the characterization of soft magnetic materials using the Epstein Frame. The proposed approach does not necessitate the incorporation of additional components into the experimental setup or the implementation of complex methodologies for the evaluation of the surface of the Epstein coil. The proposed strategy has undergone rigorous evaluation for three distinct Fe-Si commercial stripes, encompassing Non-Oriented Grain (NOG) and Oriented Grain (OG) specimens with varying thicknesses and Silicon percentages. In any case, given the independence of the proposed procedure from the material nature, it can be applied to each variety of soft magnetic material. The results obtained have been presented in the form of magnetization curves and magnetic losses across a broad spectrum of frequencies. A comparison of these results with the data provided by manufacturers has been conducted, thereby substantiating the efficacy of the proposed approach despite its apparent simplicity compared with those proposed by the standard. The findings underscore the paramount importance of implementing compensation techniques to circumvent persistent errors in the assessment of soft magnetic materials’ performance, particularly in scenarios characterized by elevated magnetic density flux values. This imperative is of primary significance for the development of accurate magnetic materials models and for the prediction of power system behavior in fault conditions.

## 1. Introduction

Soft magnetic materials play a pivotal role in electric and electronic power applications due to their capacity to be readily magnetized and demagnetized when exposed to an external magnetic field. The adoption of magnetic components grants several possible advantages and offers a range of potential benefits, which are leveraged in various applications. These include the reduction in size of inductors and transformers [1], the minimization of magnetic reluctance paths in electric motors [2], and the enhancement of coupling coefficient and electromagnetic shielding in wireless power transfer systems [3,4]. A plethora of soft magnetic materials are commercially available, including silicon–iron alloys, sintered powder cores, nanocrystalline alloys, amorphous alloys, additively manufactured silicon–iron materials, and ferrites. These materials show specific peculiarities in terms of magnetic properties (permeability, saturation, linearity, behavior at high working frequencies) [5,6,7], thermal properties (Curie temperature, behavior at high working temperatures) [8,9] and mechanical properties (mechanical strength, resistance to fatigue, sensibility to vibrations, tendency towards magnetostriction) [10,11,12,13], offering many possibilities for designers in the choice of the most suitable magnetic component for a given power application. The magnetic properties of soft magnetic materials can be evaluated with three main techniques, all standardized by the International Electrotechnical Commission (IEC) [14,15,16,17]: Epstein Frame, Single Sheet Tester (SST) and Volt-Amperometric Method (VAM). Epstein frame is applied on stripes of the material under test, which are combined in a square shape to form a closed magnetic path. SST is applied to sheets of the material to test; in this case, the magnetic path is closed by two heavy metal “C-shaped” parts called yokes, which touch the edges of the sheet. Finally, VAM is applied to toroids or other non-conventional shapes which behave as closed magnetic paths. Among them, the Epstein frame is considered as the reference international standard thanks to its accuracy. Indeed, accuracy is lower in SST due to its sensitivity to possible mechanical stress of the sheet and to the electric conductivity of the yokes; it is useful for quick tests in the industrial field since it can be applied directly to the whole manufactured sheet. Concerning instead VAM, it is recognized to be less accurate compared with the previous method since the windings for the measurement are directly wounded on the core under test, with the consequent problem related to flux leakages; it is useful for quick characterizations and to characterize real components or materials which cannot be manufactured in stripe or sheet shape (i.e., nanocrystalline materials, ferrites, sintered powder cores). For this reason, this work is focused on the Epstein frame method. The measurement setup consists of a series of four solenoid coils arranged in a square configuration. A specific quantity of stripes (a multiple of four, as recommended by the prevailing standard) of the under-test material is inserted within each solenoid to establish a closed magnetic path. The material is characterized by imposing a sinusoidal magnetic flux density (*B*) across the material. Since the *B* waveform is derived from the voltage induced in the secondary Epstein coil, it is referred to the magnetic flux detected within the entire solenoid, which includes the contributions of both material and air. For low values of *B*, the high permeability of the magnetic material results in a significant difference (typically 3 or 4 orders of magnitude) between the material and in-air magnetic flux, making the latter negligible. However, as the material approaches saturation, the two magnetic fluxes become comparable. Consequently, the in-air magnetic flux exerts a significant influence on the total magnetic flux, thereby affecting the measurement. There are several aspects in which the knowledge of the material behavior in saturation conditions is strongly required. Firstly, the accurate reproduction of the magnetization process is imperative for the development and validation of mathematical hysteresis models [18,19]. Moreover, in a power electronics system, the occurrence of short-circuit conditions due to failures of its components can lead to excessive currents, up to 20 times the nominal value [20], causing the saturation of the magnetic core. The precise depiction of the material’s behavior under conditions of saturation facilitates the simulation of the power system’s operation at elevated current levels, encompassing scenarios involving faults. This, in turn, contributes to the design of protective devices.

The IEC standard recommends two possible ways for air magnetic flux compensation. The first one consists of the addition of a compensating coil. The primary winding of the compensated coil is connected in series with the primary winding of the Epstein frame, and the secondary winding of the compensating coil is connected in anti-series with the secondary winding of the Epstein frame. The number of turns of the compensation coil is chosen in such a way that, exciting the Epstein frame without any material, the voltage measured in the secondary winding of the Epstein frame is zero. An alternative approach involves the implementation of a numerical (offline) compensation strategy, consisting of an algorithm which corrects the measured magnetic density flux. The basic compensation algorithm can be derived from the definition of the total measured magnetic flux.

(1) $$\phi =B_{stripe}S_{stripe}+B_{air}\left(S_{air}-S_{stripe}\right)=BS_{stripe}$$

where ϕ is the total magnetic flux measured in the Epstein frame, and *B* is the magnetic density flux measured according to the Epstein frame standard, which prescribes to use the material cross-section (*S_stripe_*) as the reference area. *S_air_* is the total section of the Epstein yoke. From (1), the magnetic flux density inside the material can be derived as:

(2) $$B_{stripe}={B-B}_{air}\frac{\left(S_{air}-S_{stripe}\right)}{S_{stripe}}=B-\mu_{o}H\frac{\left(S_{air}-S_{stripe}\right)}{S_{stripe}}$$

Given the established values of *B* and *H* derived from measurement, the calculation of $B_{stripe}$ is possible, provided that *S_air_* and *S_stripe_* are known. In the literature, there are two works that proposed alternatives and improvements with respect to the standard. In particular, in [21], a new Epstein frame design is proposed. Dedicated air-flux compensation coils are placed tightly bound with the magnetic field coils or integrated directly into the coil formers. These coils capture an exact equivalent of the empty space flux, ensuring that the detected secondary voltage only reflects the true polarization of the magnetic steel. Compared with the standard, this method avoids the preliminary measurement in the empty setup for the calibration of the compensation coils. In general, the main disadvantage related to the methods based on compensation coils is the necessity to incorporate an additional component, which can pose a challenge when attempting to insert it into an existing Epstein frame. In [22], a solution for numerical compensation is proposed. In particular, a methodology for deriving *S_air_* without resorting to geometric calculations is proposed. Indeed, the evaluation of *S_air_* is not trivial, given that the shape of the Epstein coil cross-section is typically a mixture of an elliptical and rectangular shape, which does not have an analytical formulation. The authors of the study posit that the calculation of *S_air_* can be performed once the material reaches a state of complete saturation. However, to perform a correct estimation of *S_air_* with this method, very high values of magnetic flux density and magnetic field strength (almost 6T and hundreds of kA/m according to the pictures presented) are required. These values are often difficult to achieve with typical Epstein frame setups, as visible in [23,24,25].

Finally, the standard IEC 60404-3:2022 [16] prescribes a numerical air flux compensation technique, which avoids the knowledge of *S_air_*. The method is born for the Single Sheet Tester (SST) characterization technique, but it can be applied to the Epstein frame setup also. However, this method requires the adoption of a non-inductive precision resistor and an initial assumption to tune, after a preliminary measurement in air conditions, a compensation factor. In this sense, the technique is not so different from the adoption of a compensating coil, since also in this case, an additional component is required in the experimental setup. For that reason, in this work, a novel computational approach for air magnetic flux compensation is presented. The primary benefit of this proposal is its complete independence from *S_air_*, requiring just a preliminary measurement (without preliminary theoretical assumptions) with the Epstein coils totally in air. Compared with the other presented methods, the proposed numerical compensation technique allows for the characterization of a material up to its saturation conditions using a standard Epstein frame setup and classic power supply equipment, resulting in a cheaper and simpler solution than the ones proposed in the literature or by the standard. The proposed approach is evaluated through experimentation on different Fe-Si alloy materials, including both Non-Oriented Grain (NOG) and Oriented Grain (OG) types. The extent to which air magnetic flux compensation impacts the outcomes of characterization for high values of magnetic flux density will be demonstrated. The proposed approach will be validated by comparing the characterization results with the manufacturer’s data. The fabrication of Fe-Si alloys in the form of stripes is a relatively straightforward process. Indeed, the Epstein characterization standard was initially developed for the purpose of evaluating this particular alloy. The Epstein technique is typically employed to also characterize Fe-Ni and Fe-Co alloys [26,27], and in general, the proposed procedure can be applied to any kind of soft magnetic material that can be manufactured in stripe shape. As will be demonstrated in the subsequent section, the mathematical procedure is entirely independent of the material nature.

## 2. Experimental Setup and Numerical Compensation of Air Magnetic Flux

### 2.1. Experimental Setup for Magnetic Characterization

**Figure 1:**
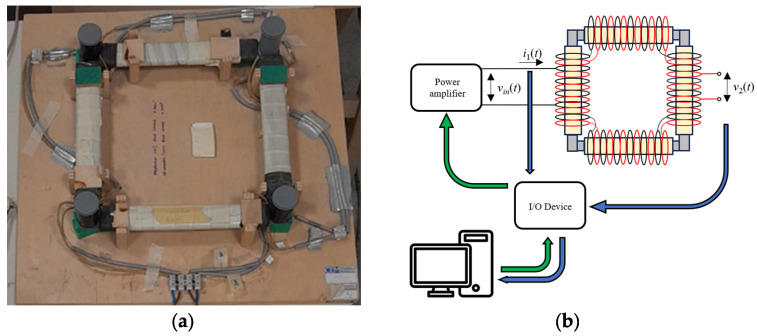
Experimental setup for magnetic characterization. (**a**) Epstein frame. (**b**) Scheme of the characterization setup. Power Amplifier: PA 100-52 (Brockhaus Messtechnik GmbH & Co KG©, Lüdenscheid, Germany). I/O (DAQ) Device: NI USB-6363 (National Instruments, Austin, TX, USA).

The setup adopted for the Epstein frame characterization is presented in Figure 1.

**Figure 2:**
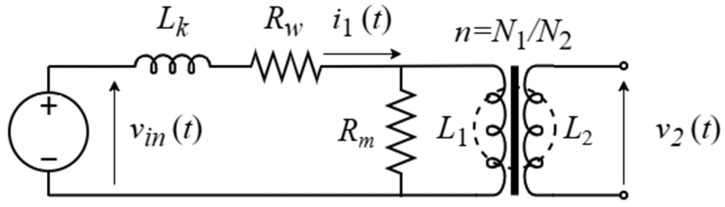
Equivalent circuit of the measurement setup.

The configuration has been meticulously engineered in strict accordance with the IEC standards established for Epstein frame characterization [14,15]. Each solenoid is composed of two windings, with a total of 840 turns per winding. The characterization is achieved through the implementation of 16 stripes, with 4 stripes positioned on each side. The materials under test consist of stripes with a length of 300 mm and a width of 30 mm. The geometric mean length of the magnetic circuit is 1.08 m, and the mean magnetic path is 0.94 m, as stipulated by the IEC standard. The equivalent circuit associated with the setup is shown in Figure 2.

Referring to Figure 2, *R_w_* denotes the winding resistance of the Epstein frame primary winding. *L*_1_ and *L*_2_ represent the magnetizing self-inductances of the two Epstein frame windings (with the material under test as the magnetic core), *L_k_* is the leakage inductance in the primary winding, while *R_m_* signifies the equivalent resistance taking into account the magnetic losses of the material under test. *N*_1_ and *N*_2_ denote the number of turns of the Epstein frame primary and secondary windings, respectively. Furthermore, *i*_1_(*t*) signifies the current in the primary Epstein coil, while *v*_2_(*t*) denotes the voltage in the secondary Epstein coil. The characterization process entails exciting the primary winding of the Epstein coil, while the secondary winding is maintained in open-circuit conditions. The current in the primary winding (*i*_1_(*t*)) and the voltage in the secondary winding (*v*_2_(*t*)) are measured. As illustrated in Figure 2, the voltage *v*_2_(*t*) exclusively refers to the material. Consequently, this methodology facilitates the precise quantification of the magnetic losses of the material, neglecting any extraneous influences that might be attributed to Joule losses within the windings. The measured current and voltage can be used to evaluate, respectively, the magnetic field intensity (*H*) and the magnetic flux density (*B*) with the Ampere Law and the Faraday Law:

(3) $$H\left(t\right)=\frac{N_{1}}{l}i_{1}\left(t\right);\;B\left(t\right)=\frac{1}{N_{2}S_{stripe}}\int v_{2}\left(t\right)dt$$

*H* and *B* can be utilized to plot the anhysteretic curve and the hysteresis loop of the material under investigation. The power losses can be calculated as the mean value of the instantaneous power across the material, evaluated during the material’s working period.

(4) $$P_{loss}(W)=\frac{1}{T}\int_{0}^{T}v_{2}\left(t\right)i_{1}(t)dt$$

where *T* is the working period. Alternatively, given that the area of the hysteresis loop is indicative of the energy dissipation (in J/m^3^) in the material for each excitation cycle, the power losses can be evaluated as follows:

(5) $$P_{loss}(W)=Vf\oint BdH$$

where *f* is the working frequency, and *V* is the material volume.

The inherent non-linear characteristics of magnetic materials complicate the implementation of a sinusoidal magnetic density flux, as required by the IEC standard. As illustrated in Figure 2, when a sinusoidal input voltage is applied to the primary Epstein coil, the resulting current *i*_1_(*t*) will generate a non-sinusoidal waveform if the material exhibits non-linear behavior. Consequently, the voltage drop in the winding resistance (*R_w_*) and the voltage in the secondary winding *v*_2_(*t*) will result in a non-sinusoidal waveform. To address this challenge, a feedback procedure has been established. At each iteration, the voltage *v*_2_(*t*) is measured and converted into a digital signal through a DAQ system (illustrated as an I/O device in Figure 1b). After conversion to *B*, a comparison is made between the resultant signal and the sinusoidal setpoint signal. The input voltage *v_in_* is updated based on the discrepancy between the measured *B* and the desired value. This adjustment is processed by a proportional-derivative (PD) controller, as depicted in (6). The updated *v_in_* is then converted into an analog signal through the data acquisition (DAQ) system and transmitted to a power amplifier.

(6) $$v_{in}^{n+1}\left(t\right)=v_{in}^{n}\left(t\right)+K_{P}\left(B_{ref}\left(t\right)-B^{n}\left(t\right)\right)+K_{D}\frac{d}{dt}\left(B_{ref}\left(t\right)-B^{n}\left(t\right)\right)$$

*n* is the iteration index, *B_ref_*(*t*) is the setpoint magnetic flux density waveform, *B^n^*(*t*) is the measured magnetic flux density at the *n*-th iteration, and *K_P_* and *K_D_* are the PD controller constants. Alternatively, controllers based on different formulations or data-driven approaches could be considered [28]. In any case, as indicated in [29], the PD controller is identified as one of the most effective solutions in terms of the number of iterations required for convergence when compared with Newton–Raphson and Broyden’s methods for frequencies of 5 Hz or higher. However, a comparison with data-driven approaches has still to be investigated. This latter approach holds particular promise to characterize materials under 5 Hz, where the conventional PD controller and Newton–Raphson method often fail to converge.

### 2.2. Numerical Compensation of Air Magnetic Flux

The magnetic flux, as previously defined, is measured in the entire Epstein coil, thereby incorporating the contributions of both material and air. In order to compensate the air magnetic flux, the following procedure is proposed. Starting from the measured *v_2_*, this latter can be written as a function of the total magnetic flux as follows:

(7) $$v_{2}=\frac{d\phi }{dt}=\frac{d}{dt}\left(\phi_{stripe}+\phi_{air}\right)=N_{2}\frac{d}{dt}\left[B_{stripe}S_{stripe}+B_{air}\left(S_{air}-S_{stripe}\right)\right]$$

As presented in (3), the calculation of *B* starting from *v_2_* is derived utilizing the $S_{stripe}$ area. Upon collecting $S_{stripe}$ in (7), the following expression is obtained:

(8) $$v_{2}=N_{2}S_{stripe}\frac{d}{dt}\left[B_{stripe}+B_{air}\frac{\left(S_{air}-S_{stripe}\right)}{S_{stripe}}\right]$$

where the quantity $B_{stripe}+B_{air}\frac{\left(S_{air}-S_{stripe}\right)}{S_{stripe}}$ represents the measured *B*. Concerning *B_air_*, it can be expressed not only like μ_o_*H*, but also according to the Faraday Law. Specifically, naming as *v_air_* the voltage measured in the empty Epstein secondary coil, *B_air_* can be expressed as follows:

(9) $$B_{air}=\frac{1}{N_{2}S_{air}}\int v_{air}\left(t\right)dt$$

Concerning *B_stripe_*, it can be written, by definition, as:

(10) $$B_{stripe}=\mu_{o}\left(H+M_{stripe}\right)=B_{air}+\mu_{o}M_{stripe}$$

where *M_stripe_* is the magnetization of the material. In this way, the measured *B* can be rewritten as:

(11) $$B=\mu_{o}M_{stripe}+B_{air}\frac{S_{air}}{S_{stripe}}$$

From (11), remembering the double possibility to express *B_air_* (as μ_o_*H* and as reported in (9)), the expression of *B_stripe_* can be finally derived as:

(12) $$B_{stripe}=\mu_{o}M_{stripe}+B_{air}=B-B_{air}\frac{S_{air}}{S_{stripe}}+\mu_{o}H=B-\frac{\phi_{air}}{S_{tripe}}+\mu_{o}H$$

where $\phi_{air}=\frac{1}{N_{2}}\int v_{air}\left(t\right)dt$. As can be seen, this procedure does not require the knowledge of *S_air_*. This makes it an easy tool for numerical air magnetic flux compensation, where a mere preliminary measurement in air is required for its application. As can be seen, the quantity ($-\frac{\phi_{air}}{S_{tripe}}+\mu_{o}H$), which realizes the compensation, is totally independent of specific features of the material under test: it means that this technique can be theoretically applied to each kind of magnetic material. In the subsequent section, the impact of this compensation on the characterization of diverse NOG and OG materials will be demonstrated.

## 3. Results

In this study, three distinct Fe-Si alloy commercial materials were examined. Their main features are outlined in Table 1.

**Figure 3:**
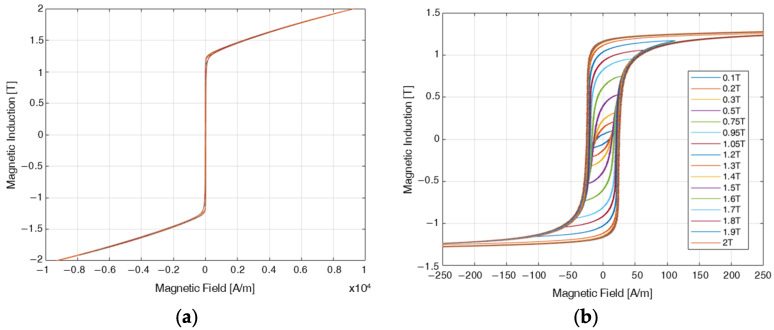

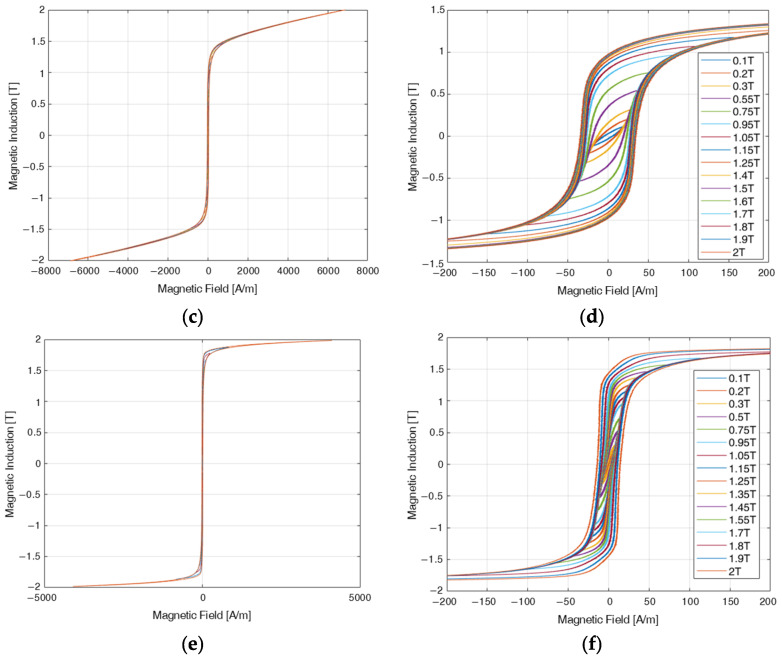
Characterization of materials up to 2 T without air flux compensation. (**a**) 10JNEX900. (**b**) 10JNEX900 zoom. (**c**) 10JNRF950B. (**d**) 10JNRF950B zoom. (**e**) H20GK075. (**f**) H20GK075 zoom.

All the materials have been characterized in their rolling direction, which is the same direction in which the manufacturer has reported its own data. The following stacking factors have been considered in the characterization of these materials: 0.94 for the NOG grades and 0.96 for the OG grade. A thermocouple has been applied to the surface of the specimen to ensure its temperature remains within the range recommended by the IEC standard (23±5 °C). A DP10013 voltage probe by Micsig (Accuracy ±2%) was employed to derive the magnetic flux density (*B*), and a CP6030 current probe by Siglent (Accuracy ±1%) was utilized to derive the magnetic field strength (*H*). The purpose of the measurements that will be shown in this section is the derivation of the anhysteretic curve of the material, to observe how it is influenced by the adoption of the proposed numerical compensation technique. To do so, a large number of hysteresis cycles is required, from the lower magnetic flux density values up to the saturation. A preliminary characterization without applying compensation techniques has been conducted at 5 Hz (quasi-static conditions). The results are presented in Figure 3.

As can be seen, the behavior of the three materials is quite different due to differences in terms of Silicon percentage and grain orientation. The adoption of materials with different properties allows for demonstrating the general validity of the proposed compensation technique. According to Figure 3, a magnetic density flux value of 2 T can be attained in all the materials under consideration; note that this value is not consistent with the magnetic saturation values declared by the manufacturer and reported in Table 1. Therefore, it can be concluded that a compensation procedure is necessary to prevent errors in the characterization of the materials.

**Figure 4:**
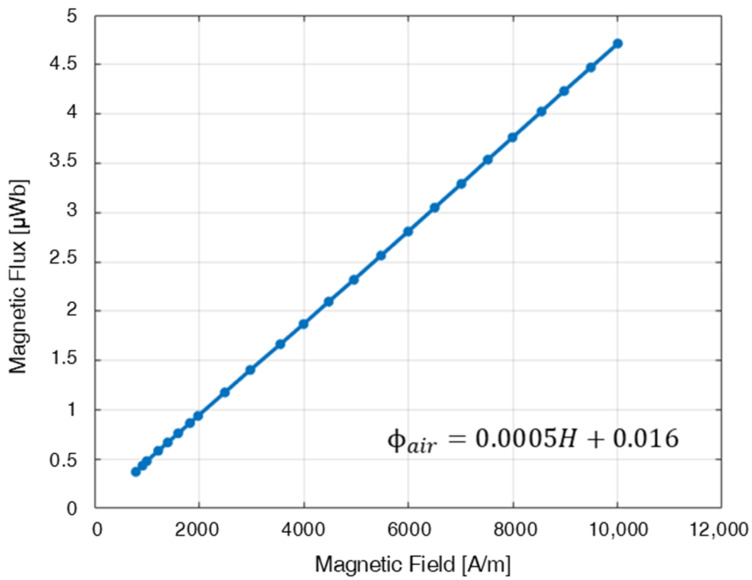
Air magnetic flux versus magnetic field intensity.

As delineated in the previous section, the proposed compensation procedure requires a preliminary measurement of the magnetic air flux. Given that the maximum magnetic field intensity recorded in the characterization depicted in Figure 3 is 10 kA/m, the measurement in air was also conducted up to this value. The results are presented in Figure 4. The alignment of the measured points, as predicted by the theory, demonstrates a notable degree of robustness and minimal sensitivity to noise.

**Figure 5:**
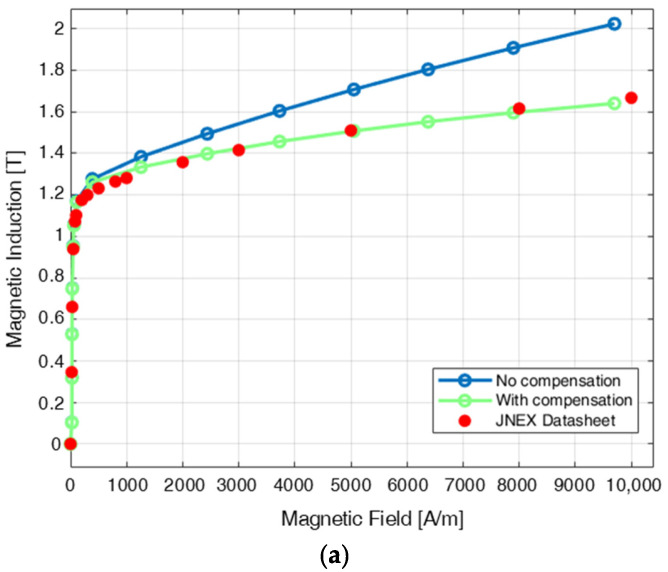

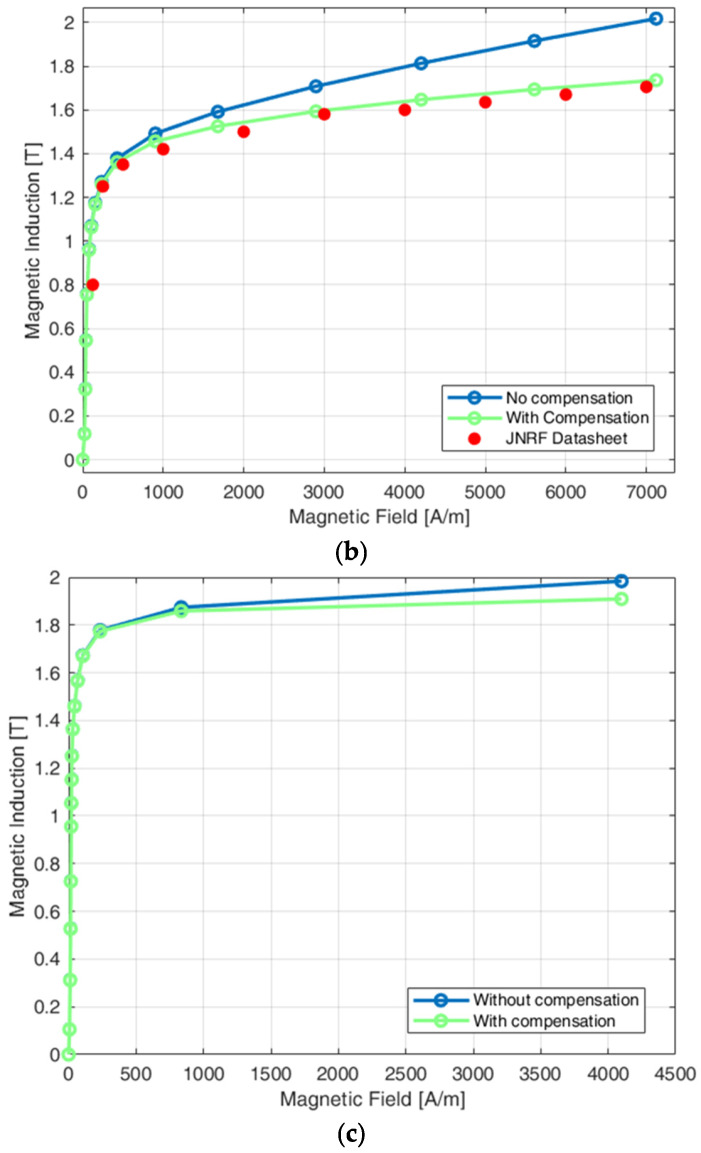
Comparison of the magnetization curve obtained with or without in-air magnetic flux compensation. (**a**) 10JNEX900 (MSE = 0.0090 between green curve and red points). (**b**) 10JNRF950B (MSE = 0.0099 between green curve and red points). (**c**) H20GK075.

The regression line derived from in-air measurements and shown in Figure 4 is used to apply the compensation technique presented in (12). It is noteworthy that the magnetic field intensity values depicted in Figure 3 and Figure 4 are considerably lower in comparison to the ones required in [22], so they can be easily attained with standard experimental apparatus. The comparison between characterization results achieved with or without the air flux magnetic compensation is performed both in terms of magnetization curve and losses. The magnetization curve has been derived by interpolating the peak values of *H* and *B* of all the hysteresis loops measured at *f* = 5 Hz. At this frequency, the material’s behavior can be considered quasi-static from a magnetic perspective. Consequently, the curve resulting from the interpolation of the hysteresis loops can be regarded as analogous to the DC magnetization curve. The magnetization curves derived from Epstein measurements, whether taken with or without the application of the in-air flux compensation, have been compared. For 10JNEX900 and 10JNRF950B grades, the DC magnetization curves declared by the manufacturer are available [30,31]. They have been implied to validate the proposed compensation technique. The results are presented in Figure 5 and summarized in Table 2.

**Figure 6:**
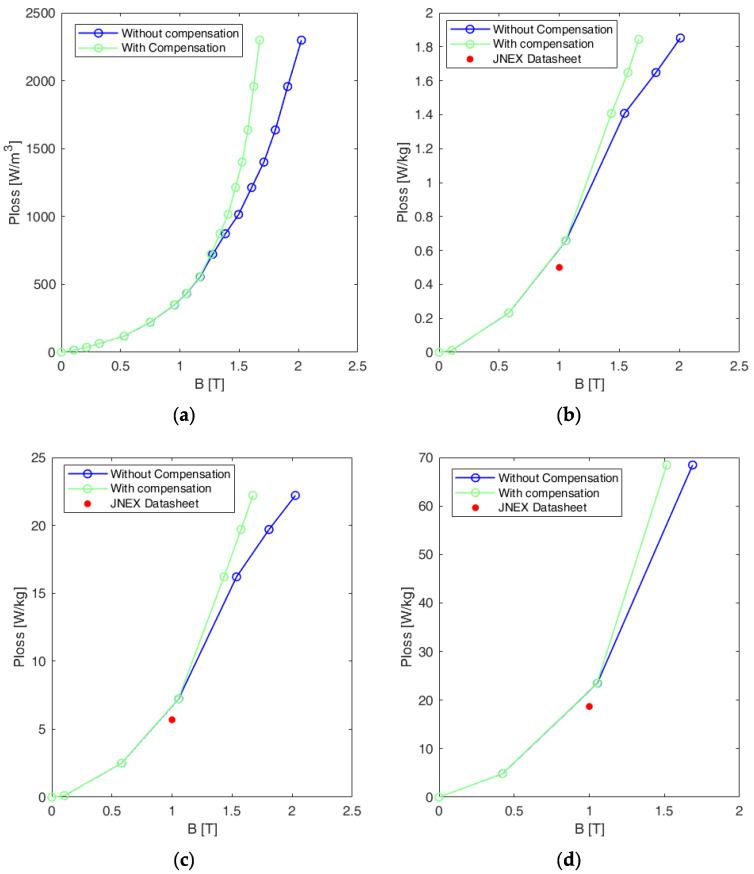
Comparison of measured losses obtained with or without in-air magnetic flux compensation for 10JNEX900 grade. (**a**) 5 Hz (**b**) 50 Hz. (**c**) 400 Hz. (**d**) 1000 Hz.

**Figure 7:**
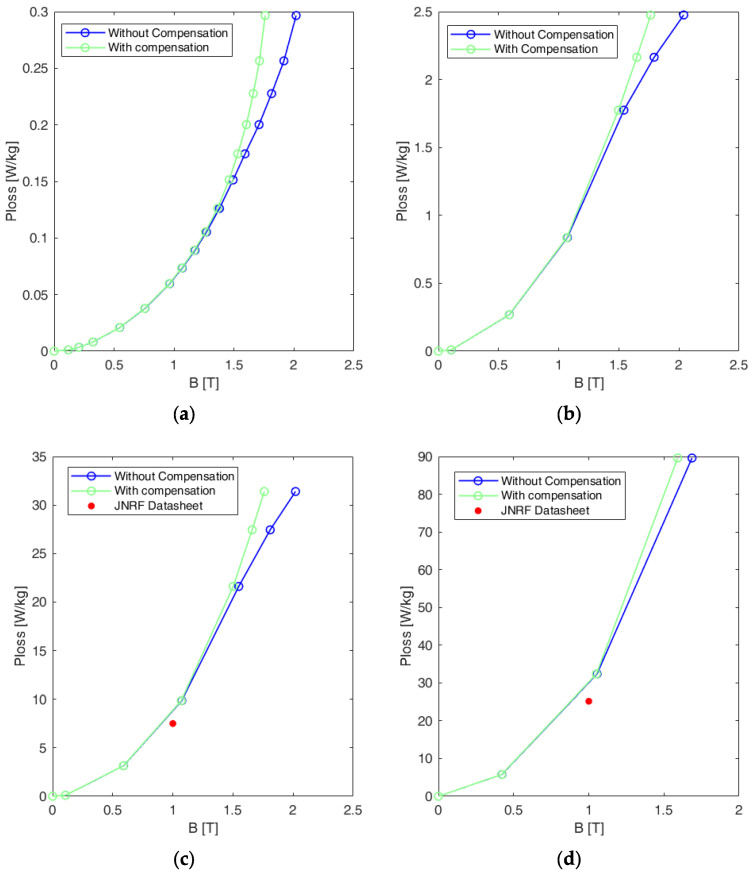
Comparison of measured losses obtained with or without in-air magnetic flux compensation for 10JNRF950B grade. (**a**) 5 Hz (**b**) 50 Hz. (**c**) 400 Hz. (**d**) 1000 Hz.

**Figure 8:**
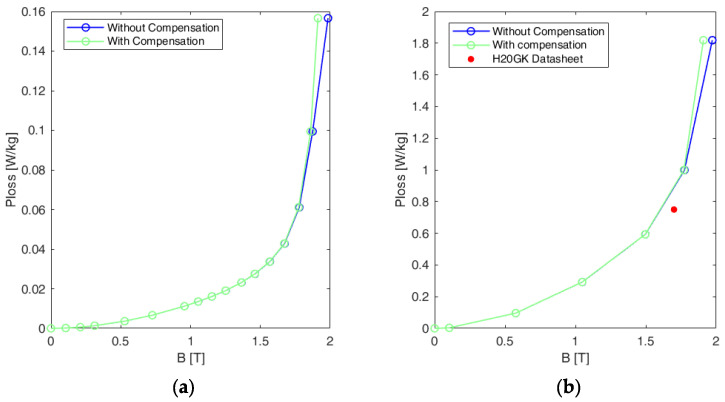

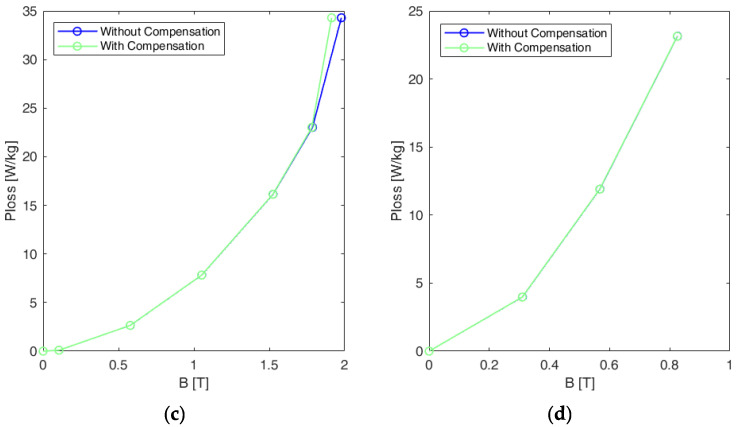
Comparison of measured losses obtained with or without in-air magnetic flux compensation for H20GK075 grade. (**a**) 5 Hz (**b**) 50 Hz. (**c**) 400 Hz. (**d**) 1000 Hz.

Several considerations can be made in light of the results depicted in Figure 5 and Table 2. Firstly, it is evident that the in-air magnetic flux exerts a more substantial influence on NOG material, while its effect on OG material is considerably less pronounced. This phenomenon can be attributed to two primary factors. The first is that OG exhibits a higher magnetic permeability compared to NOG material. Consequently, the magnetic field intensity required to attain a similar magnetic flux density is reduced, resulting in a lower air magnetic flux. Additionally, as demonstrated in Table 1, OG possesses a greater thickness, which consequently results in a higher *S_stripe_*. According to (12), this higher stripe thickness serves to mitigate the impact of ϕ*_air_*. Concerning the NOG material, it is evident that compensation is imperative to circumvent substantial errors in characterization in proximity to saturation. In this instance, the predominant effect is observable in the 10JNEX900 grade, given its reduced magnetic saturation, attributable to its elevated silicon content (6.5%). In any case, as presented in Table 2, the proposed compensation technique, despite its simplicity compared with the methods proposed in the literature or prescribed by standards, allows for eliminating the measurement error in the saturation region, aligning the measured values with the ones declared by the manufacturer. The comparison in terms of losses is presented in Figure 6, Figure 7 and Figure 8, which compare the values achievable with and without in-air magnetic flux compensation. Four distinct frequencies have been evaluated for the characterization: 5 Hz, 50 Hz, 400 Hz, and 1000 Hz. When available, a comparison has also been made between the measured losses and the data provided by the manufacturer [30,31,32].

A significant disparity becomes evident in terms of losses for NOG grades approaching saturation conditions. In this instance as well, the difference is limited in the OG material, consistent with the rationales previously expounded. For the H20GK075 grade, losses at 1000 Hz are shown only up to 0.8 T due to the limitations of the adopted power supply system. It is noteworthy that, due to the double thickness of OG material in comparison with NOG grades under the same voltage condition, only half of the magnetic flux density can be attained, as dictated in Faraday’s Law (3). The data provided by the manufacturer refer to magnetic flux density values that are considerably away from saturation. Consequently, it is not possible to discern any differences in terms of losses measured regarding the application of the proposed compensation procedure. A slight overestimation is observable from the measurements in comparison with the datasheet (in a range from 5% to 15%). This discrepancy could be attributed to the effective dimensions of the stripes, as the nominal values of length and cross-section, as declared by the manufacturer, have been adopted in the presented procedure. In addition, the observed variation may be attributable to the statistical measurement uncertainty. In any case, the discrepancy is negligible. These figures demonstrate the significant impact of compensation on the characterization of magnetic materials, particularly in the context of NOG grades.

## 4. Conclusions

The presented paper proposes a numerical compensation procedure that has the potential to suppress the contribution of the air magnetic flux in the characterization of soft magnetic materials using the Epstein frame method. The primary benefit of the proposed strategy is that it does not necessitate the incorporation of additional components into the experimental setup or the implementation of complex methodologies for the evaluation of the surface of the Epstein yoke. The strategy has undergone rigorous evaluation on three distinct Fe-Si stripes, encompassing both non-oriented grain and grain-oriented grades, with varying thicknesses and silicon percentages, to demonstrate the general validity of the proposed approach, which could also be extended to any other kind of soft magnetic material. The results obtained from the experimental study have been thoroughly analyzed in terms of the magnetization curve and magnetic losses across a broad spectrum of frequencies. These findings have been meticulously compared with the data provided by manufacturers to ensure the validity and reliability of the results. The findings demonstrate a notable similarity between the magnetization curves measured on the tested grades and the curves provided by manufacturers, validating the approach’s effectiveness despite its apparent simplicity. A comparison of losses across various magnetic flux density values and frequencies has been conducted. A significant discrepancy has been observed in the magnetization curves and losses of NOG samples as they approach saturation conditions. This discrepancy can be attributed to the reduced thickness and diminished permeability of the NOG samples in comparison to the OG sample. The reduced thickness and permeability of the NOG samples result in an augmented impact of the in-air magnetic flux. In summary, the proposed approach was found to be an efficient and easily implementable method for accurately and comprehensively characterizing soft magnetic materials. In this sense, it is a valuable alternative to the approaches proposed by the standard. The findings of the study indicate the paramount importance of implementing compensation techniques to circumvent the occurrence of persistent errors in the assessment of soft magnetic materials’ performance, particularly in scenarios involving high magnetic density flux values.

## Figures and Tables

**Table 1:** Main features of materials under test.

Grade	Manufacturer	Thickness	Nominal Saturation
10JNEX900 (NOG)	JFE Steel, Tokyo, Japan	0.1 mm	1.6 T
10JNRF950B (NOG)	JFE Steel, Tokyo, Japan	0.1 mm	1.8 T
H20GK075 (OG)	Guangdong Hongwang Metal New Material Co., Ltd., Guangdong, China	0.2 mm	1.9 T

**Table 2:** Main parameters deduced from magnetization curves comparison (Figure 5).

Grade	Peak B [T] Not Compensated	Peak B [T] Compensated	Peak B [T] Datasheet	MSE Compensated—Datasheet
10JNEX900	2.044	1.627	1.668	0.0090
10JNRF950B	2.009	1.717	1.705	0.0099
H20GK075	1.984	1.909	Not available	Not available

## Data Availability

The original contributions presented in this study are included in the article. Further inquiries can be directed to the corresponding author.
